# An Interactive Internet-Based Continuing Education Course on Sexually Transmitted Diseases for Physicians and Midwives in Peru

**DOI:** 10.1371/journal.pone.0019318

**Published:** 2011-05-09

**Authors:** Fredy A. Canchihuaman, Patricia J. Garcia, Stephen S. Gloyd, King K. Holmes

**Affiliations:** 1 School of Public Health and Administration, Universidad Peruana Cayetano Heredia, Lima, Peru; 2 Departments of Global Health and Epidemiology, University of Washington, Seattle, Washington, United States of America; 3 Center for AIDS and STD, University of Washington, Seattle, Washington, United States of America; 4 Department of Medicine, University of Washington, Seattle, Washington, United States of America; University of Pittsburgh Medical Center, United States of America

## Abstract

**Background:**

Clinicians in developing countries have had limited access to continuing education (CE) outside major cities, and CE strategies have had limited impact on sustainable change in performance. New educational tools could improve CE accessibility and effectiveness.

**Methodology/Principal Findings:**

The objective of this study was to evaluate an interactive Internet-based CE course on Sexually Transmitted Diseases (STDs) management for clinicians in Peru. Participants included physicians and midwives in private practice drawn from a census of 10 Peruvian cities. The CE included a three-hour workshop for improving Internet skills, followed by a 22-hour online course on STD-syndrome-management, with subsequent educational support. The course used case-based clinical vignettes tailored to local STD problems. Knowledge and reported practices on STD management were assessed before, immediately after and at four months after completion of the course. Statistical analysis included parametric tests-linear regression multivariate analysis, paired t-test and repeated measures ANOVA using SPSS 14.0. Of 1,071 eligible clinicians, 510 agreed to participate, as did an additional 132 public sector clinicians. Of these 642 participants, 619 (96.4%) completed the course, and 596 (96.3%) took the four-month follow-up evaluation. Physician and midwife scores improved from 64.2% correct answers on the pre-test to 77.9% correct on the four-month follow-up test (p<0.001). Most participants (95%) found the online course useful for their work needs. Self reported STD management practices did not change.

**Conclusions/Significance:**

Among physicians and midwives in Peru, an Internet-based CE course was feasible, acceptable with high participation rates, and led to sustained improvement in knowledge at four months. Further studies are needed to test it as a model for improving the training of physicians, midwives, and other health care providers.

## Introduction

Continuing education (CE) for health care workers is required by professional credentialing, governmental and licensing agencies and is available in many developed countries, but in developing countries, accessibility to those programs is limited, especially outside major urban settings. In addition, traditional, didactic CE programs for health professionals have shown modest impact on sustained improvement in knowledge, health provider practices or patient outcomes[1], [2].

An alternative to traditional CE is Internet-based CE (I-CE). A number of advantages of I-CE have been proposed including the use of complex information, real-time interactive links, images, audio, and video; flexibility in location and time; potential for reinforcement through continuous availability; adaptability to adult learning approaches; potential low cost; and accessibility to providers outside major urban centers[3], [4], [5], [6].

In recent years, effectiveness of I-CE has improved by designing courses based upon educational theory and by including new educational tools such as case scenarios or clinical vignettes[4], [7], [8], [9]. For example, courses based upon situated learning theory (learning in the context of the interaction between the participants and their environment) and involving cognitive processes – decision-making, reasoning, and problem-solving – can help develop skills in medical practice[10], [11].

In Peru, as in other developing countries, the telecommunications infrastructure has improved rapidly[12] and Internet access is widely available. However, use of information and communication technologies in health remains limited[13], [14], [15]. Evidence for feasibility, acceptability, and effectiveness of I-CE for health care providers' training is largely lacking in our countries; therefore development and evaluation of such programs is warranted[13].

We designed and implemented an interactive, I-CE course on syndromic management of sexually transmitted diseases (STDs) using cognitive-educational theories. This study evaluates feasibility, acceptability, and impact of this course on the knowledge and reported STD management practices of participants.

## Methods

### Study design and population

The study was designed as a pre-post evaluation of the I-CE course, with repeated measures to compare knowledge and self-reported practices at baseline (before the course), immediately after, and at four-months after completion of the course.

**Figure 1:**
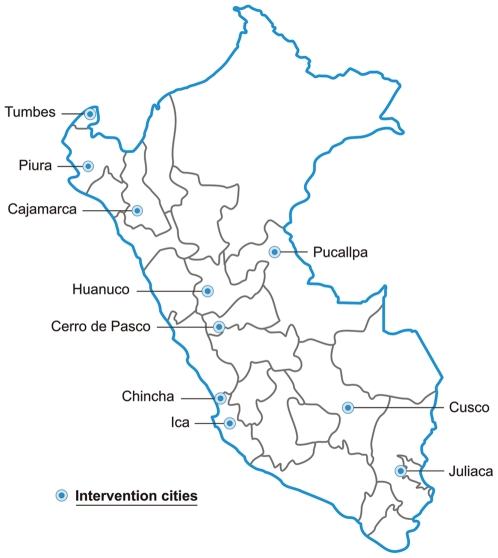
Map of Peru with the location of the 10 intervention cities (each city has more than 50,000 inhabitants).

The I-CE course was developed as a training component for the Urban Community-Randomized Trial of STD Prevention in Peru (The PREVEN study)[16]. The training component and the evaluation were implemented between August 2005 and March 2006 in the 10 intervention cities included in PREVEN, representing coastal, jungle and Andean regions of Peru. (Figure 1).

Based on a census of physicians and midwives in private practice done in 2003 and updated yearly[17], [18], we sent invitations to all of them to participate in the training program. The invitation described the nature of the course (internet-based), and the inclusion of initial training in use of the Internet tools. Advertisements were also posted in health centers.

### Course design, content and certification

The course was designed in a user-friendly, modular platform using an open-source programming language, Hypertext Preprocessor (PHP). The program included not only the educational content but also an administrator module, a database to store participants' data (demographics, scores, etc) and a report generator.

The course was developed by a team from the Unit of Epidemiology and STD/HIV, Universidad Peruana Cayetano Heredia (UPCH), who wrote the national guidelines for STD management for the Ministry of Health of Peru, and had extensive experience in STD management training. The course was based on World Health Organization[19] guidelines for syndromic management of STDs, and on the Peruvian National Guidelines for STD management. Content addressed four STD syndromes (vaginal discharge, urethral discharge, pelvic inflammatory disease, and genital ulcer disease). Additional components of the course included learning materials and links to STD resources on the Internet, materials for the patient, opportunities to “ask the expert”, and responses to frequently asked questions (FAQ). Further feedback and educational support were provided through post-course consultations via e-mail and summarized on the FAQ section. Course completion averaged 22 hours duration over a three-week period.

Several case scenarios (clinical vignettes) were used to introduce each of the four STD-syndromes and to illustrate diagnosis, treatment, partner's treatment, promotion of condom use, and patient follow-up. Each case-scenario included an image and brief history from the clinical vignette. Sequential questions, addressing common mistakes in STD management[20], were posed after each vignette. (Appendix S1). Feedback was provided for each answer, whether answered correctly or incorrectly. Additional windows offered in-depth discussions.

The course presented an overview of history taking and physical examination, and counseling regarding risk behavior. (Appendix S2).

The course was evaluated by the Peruvian College of Medicine and was given 1.5 continuing medical education (CME) credits which could be used towards the renewal of medical licenses (10 CME credits every five years are required for renewal of medical licenses). UPCH also accredited 1.5 credits for the course, which was helpful for midwives. To obtain the credits, participants had to complete both pre- and post-tests and required a post-test score of more than 60 out of 100 points.

### Course piloting and implementation

The course was piloted in Lima with a group of 15 physicians and midwives. Their feedback guided improvements in the design of the course and clarity of the questions, and allowed assessment of the time needed to complete of the course.

In each of the 10 cities, we next invited all interested clinicians to attend a three-hour workshop in Internet cafes rented for the purpose. The first hour provided training in use of the Internet and in completing the pre-test. During the remaining two hours, participants began reviewing the course Website. Each module could be completed during one or more sessions and in different places – participants were allowed to skip forward and return to the modules. The course was free and available for CME credits over a period of eight weeks. For each participant, a post-test was scheduled three weeks after beginning the course, and a follow-up test was scheduled four-months post-training.

### Course evaluation

The training was evaluated through comparing pre-test knowledge with post-tests knowledge. The pre-test, post-test of knowledge and follow-up tests were the same, but the question order was altered. The test was structured with 22 questions and vignettes, each with one correct answer. Five additional questions about self-reported practices were included in the pre-test and follow-up test. The areas queried were frequency of use of algorithms for syndromic management of patients with STDs, use of “the four Cs” (counseling, condom promotion, compliance with treatment, and contact tracing/partner treatment) during patients' consultations, giving information to patients about their STDs, giving referral cards for patients' partners, and giving treatment for patients' sexual contacts. These questions were validated by experts and by 15 physicians and midwives for clarity of the language and content relevance.

Both pre-test and post-test were taken on the Internet. For the follow-up test participants were visited by a project worker and were given a paper-based test. After the post-test, a voluntary online survey assessed user satisfaction, acceptability, relevance of the course, and software/program performance.

### Data entry and analysis

The scores and answers from pre and post-tests were maintained in a data base (My SQL). We compared mean percentages of correct answers on pre-, post-, and follow-up tests by t-test for paired comparisons and ANOVA test for repeated measures both for clinicians who completed training, and for a subset who did not receive training. Differences between subgroups in the change of scores were assessed by t-test for independent samples. Analyses of factors associated with gain in knowledge included parametric tests (linear regression multivariate analysis, t-test and repeated measures ANOVA). Data analysis used SPSS 14.0 (SPSS Inc, Chicago, Ill) software.

The Institutional Review Boards of UPCH and the University of Washington approved the project which qualified for exemption of consent. The study evaluated overall effectiveness of the course, posed no risk to the participants, and data were analyzed anonymously. Participants received no financial incentives.

## Results

### Characteristics of the study population

Of 1071 eligible private practice physicians and midwives identified in the 10 cities, 510 (47.6%) agreed to participate and took the pre-test. Additionally, 132 physicians and midwives not in private practice participated.

Table 1 summarizes characteristics of course participants and non-participants. Physician participants averaged 41.5 years of age and 13 years of practice, 72.1% were male, and 47.5% were general practitioners; 98.2% reported seeing STD cases, and 61.2% had previously attended didactic workshops on STD management and were members of the intervention trial network (the PREVEN network). Midwives averaged 38 years of age and 11 years of practice; 84.7% were female; 93.1% reported seeing STD cases and 58.6% were PREVEN network members.

**Table 1:** Characteristics of physician and midwife participants and non-participants in the STD I-CE course.*

	Course participants				Course non-participants	
	(N = 642)				(N = 527)**	
	Course users		Course non-users			
	(N = 619)		(N = 23)			
Characteristics	Physicians	Midwives	Physicians	Midwives	Physicians	Midwives
	n = 286	n = 333	n = 15	n = 3	n = 307	n = 220
Age, years mean ±SD	41.5 (10.7)	37.9 (8.6)	45.1 (12.6)	35.3 (3.2)	45.5 (12.0)	37.3 (7.5)
Male gender, n (%)	208 (72.7)	51 (15.3)	10 (66.7)	1 (33.3)	238 (77.5)	45 (20.5)
Physicians' specialty, n (%)						
General medicine (no specialty)	134 (47.5)		4 (26.7)		141 (46.1)	
Gynecology	69 (24.4)		7 (46.7)		59 (19.3)	
Internal medicine	19 (6.7)		2 (13.3)		24 (7.8)	
Other	60 (21.3)		2 (13.3)		82 (26.8)	
Length of practice, years mean ±SD	13.0 (10.1)	11 (7.6)	15.4 (11.9)	6.3 (1.2)	16.6 (11.4)	10.7 (6.3)
Private practice, n (%)	242 (84.6)	246 (73.9)	15 (100)	3 (100)	307 (100)	220 (100)
Practice sector†, n (%)						
Private institutions	161 (46.3)	58 (17.5)	12 (80.0)	-	140 (45.8)	51 (23.2)
Social Security	82 (23.7)	30 (9.1)	5 (33.3)	-	72 (23.5)	9 (4.4)
Ministry of Health	177 (50.9)	155 (46.8)	7 (46.7)	2 (66.7)	155 (50.7)	74 (36.1)
Military	23 (6.6)	5 (1.5)	-	-	23 (7.5)	4 (2.01)
Universities	38 (13.5)	57 (17.2)	4 (26.7)	1 (33.3)	35 (11.4)	15 (6.8)
Other	27 (9.4)	83 (24.9)	1 (6.7)	1 (33.3)	28 (9.2)	73 (35.6)
PREVEN Network members††, n (%)	175 (61.2)	195 (58.6)	13 (86.7)	3 (100)	174 (56.7)	122 (55.5)

*Numbers may not add to total because of missing data.

**Of the 561 total number of course non participants (private physicians and midwives who did not agree to participate) information was available only on 527 of them.

† Many physicians and some midwives practiced in more than one sector.

†† PREVEN Network members were physicians and midwives who joined the PREVEN Network after completing a course and passing a test on STD and contraceptive management approximately a year prior to this study.

Physician non-participants had an older mean age than participants (45.5 SD±12.0 versus 41.5 SD±10.7), and had a longer mean duration of practice (16.6 SD±11.4 versus 13.0 SD±10.1 years). Midwives who did not participate were more likely than participants to be male (20.5% versus 15.3%).

**Figure 2:**
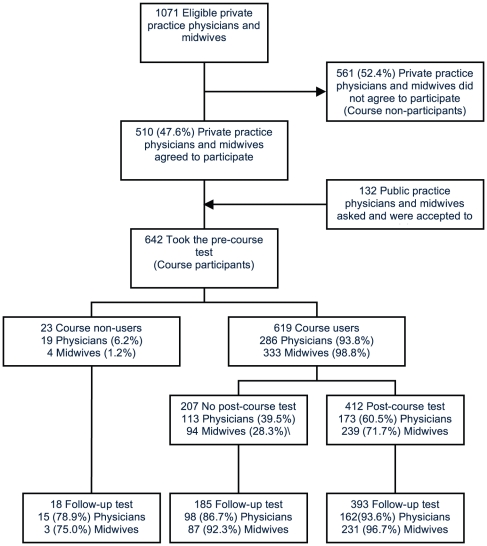
Participation flow of the Internet-based CE intervention.

Of the 642 course participants, 619 (96.4%) completed the course (course-users). Among these 619, 412 (66.6%) took the post- test; 394 (95.6%) passed the test, receiving CE credits. Among the 642 course participants, 596 (92.8%) took the four-month follow-up test. Of the 46 persons who did not take the follow-up test 4 (8.7%) were on vacation, 19 (41.3%) had moved to another town, and 23 (50.0%) were otherwise unavailable. (Figure 2).

### Knowledge of STDs

Physicians' mean total scores improved from 65.9% correct answers at pre-test to 78.0% correct at four month follow-up test (p<0.001). Midwives improved from 62.8% to 77.8% (p<0.001). (Table 2). Further unpaired analysis comparing pre-test and follow-up test did not change these associations.

**Table 2:** Participants' knowledge scores and self-reported practices of syndromic management of STDs.

		Pre-test	Follow-up test	
	n†	(baseline)	(after four months)	P value
**Knowledge scores** *		Mean (SD)	Mean (SD)	
**Physicians & midwives**	596	64.2 (12.7)	77.9 (12.5)	<0.001‡
**Physicians**	275	65.9 (12.8)	78.0 (12.3)	<0.001‡
**Midwives**	321	62.8 (12.4)	77.8 (12.6)	<0.001‡
**Practice self-reported indicators** §		Percentages	Percentages	
**Midwives**				
Use of algorithms for syndromic management of patients with STDs	296	90.2	89.5	0.888£
Give information to patients about their STDs	296	98.3	99.0	0.687£
Use of “the four Cs” (counseling, condom promotion, compliance with treatment, and contact tracing/partner treatment) during patients' consultations	297	96.0	95.3	0.832£
Give referral cards for patients' partners	292	68.5	46.2	<0.001£
Give treatment for patients' sexual contacts	297	88.9	88.6	1.000£
**Physicians**				
Use of algorithms for syndromic management of patients with STDs	238	89.5	92.0	0.307£
Give information to patients about their STDs	237	97.0	97.5	1.000£
Use of “the four Cs” (counseling, condom promotion, compliance with treatment, and contact tracing/partner treatment) during patients' consultations	237	94.9	94.1	0.815£
Give referral cards for patients' partners	235	51.1	46.0	0.257£
Give treatment for patients' sexual contacts	235	88.9	87.7	0.749£

*Percentage of correct answers (0–100). The pre-course test, post-course test and follow-up test contained the same questions, but presented in different sequences.

† Only includes participants who completed tests at both baseline and outcome.

‡ Paired samples t test.

§ Questions asked frequency of each practice performed. Percentage prevalence indicates practices that were reported as “often” or “very often”. The pre-test and follow-up test contained the same questions.

£ MacNemar test.

For the 393 physicians and midwives who took all three tests (pre, post, and follow-up tests), mean post-course scores (81.4) and follow-up scores (78.8) both surpassed mean pre-test scores (65.0) (p<0.001 for both comparisons). The decline in follow-up test scores compared to post-test scores was modest but significant (p<0.001).

Among 23 non-course users, 18 (15 physicians and 3 midwives) took both the pre and follow-up test. Their average scores improved only slightly from 72.0 for the pre-test to 76 for the follow-up test (p = 0.29).

In bivariate analysis (Table 3), factors associated with greater improvements in knowledge scores included female gender, being a midwife, attending the initial training workshop, participating in the course, and not already being a PREVEN Network member (p<0.05 for all comparisons,).

**Table 3:** Bivariate and multivariate analysis of factors associated with improvements in knowledge of syndromic management of STDs.

		Bivariate		Multivariate§	
Factors		Percentage Increase in			
		knowledge score			
		from pre-course test to follow-			
		(up test)			
	n*	Mean (SD)	P value†	Coefficient	P value
**Overall change from baseline**	596	13.8 (16.2)	<0.001		
Age					
≤30	122	13.5 (16.0)		Ref	
31–40	211	13.7 (15.4)		2.1	0.269
41–50	170	14.0 (15.0)		2.7	0.178
>50	93	13.2 (19.9)		3.1	0.189
**Gender**					
Female	342	15.2 (16.0)	0.006	Ref	
Male	254	11.5 (16.2)		−3.1	0.022
**Profession**					
Midwives	321	15.0 (16.1)	0.031		
Physicians	275	12.1 (16.1)			
**Pre-course workshop attendance**					
No	124	8.4 (17.9)	<0.001	Ref	
Yes	472	15.0 (15.4)		5.8	<0.001
**Post-course test**					
No	203	13.4 (16.5)	0.789		
Yes	393	13.8 (15.9)			
**Course participation**					
No	18	4.0 (15.6)	0.010		
Yes	578	14.0 (16.1)			
**PREVEN members**					
No	220	16.0 (16.4)	0.007	Ref	
Yes	376	12.3 (15.9)		−3.7	0.043
**Self rank of Internet skills**					
1	7	9.1 (11.4)	0.066‡		
2	62	13.1 (19.9)			
3	325	15.3 (15.9)			
4	73	10.6 (15.7)			
5	129	11.8 (14.9)			
**Private practice**					
No	111	15.9 (15.8)	0.101	Ref	
Yes	485	13.1 (16.2)		0.232	0.914

*Numbers may not add to total because of missing data.

† Student's t-test in conjunction with Levene test for equal variances.

‡ One-way ANOVA, for self rank of Internet skills, 1 =  lowest, 5 =  highest.

§ Number of observations = 596. Factors eliminated from the model were profession, post-course test, course participation, and self rank of Internet skills.

In the multivariate analysis (Table 3), the most important factors positively associated with improvement in knowledge score were attendance to the initial training workshop, not being a PREVEN member, and being female (p<0.05).

### Self-reported STD syndromic management practices

Among all participants, there were no statistically significant differences in self- reported STD management practices between the pre-test and the follow-up test except for giving card referrals for partners or sexual contacts, which for midwives fell from 68.5% in the pre-test to 46.2% follow-up test. (Table 2).

### Participants' satisfaction

Table 4 summarizes course satisfaction and acceptability among the 412 who completed the post-test; 388 (94.2%) rated the course “very useful” for their job needs and/or their professional effectiveness.

**Table 4:** Participants' satisfaction with the STD Internet-based CE course.

Statements regarding course	Physicians	Midwives
	(n = 173)	(n = 239)
	n (%)	n (%)
The primary objective of this course was substantially or fully achieved*	172 (99.4)	237 (99.2)
The course program was very useful in addressing on-the-job needs and/or improving professional effectiveness†	165 (95.4)	223 (93.3)
Based on the experience with this online course, I definitely would be interested in participating in other online courses in the same field‡	165 (95.4)	221 (92.5)
Based on the experience with this online course, I definitely would recommend it to my colleagues‡	164 (94.8)	226 (94.6)
This type of course was good or excellent compared to other web courses§	168 (97.1)	234 (97.9)

Participants rated the course experiences using the following options:

*1 = not achieved, 2 =  achieved, 3 =  substantially achieved, and 4 =  fully achieved.

† 1 = not useful at all, 2 =  not useful, 3 =  somewhat useful and 4 =  very useful.

‡ 1 = definitely no, 2 = probably no, 3 = probably yes, and 4 = definitely yes.

§ 1 =  poor, 2 =  average, 3 = good, and 4 = excellent.

Physicians were also asked to assess the following specific elements of the course: clarity, navigability, utility of images, and interactivity; mean scores of each exceeded 4.5 by Likert scale (1 = strongly disagree to 5 = strongly agree) for the statements that the course was clear and easy to understand; easy to use; that images, photos, and graphs enhanced understanding; and that the Web site was interactive.

When asked whether a course in text format (i.e., using written materials) would be preferred over an online course 305 (74%) “disagree or totally disagreed”. Physicians reported willingness to participate in a similar internet-based course in the future and reported they would recommend participation to colleagues.

Written comments about the course were consistently favorable, and many requested additional courses and ongoing updates, including more images, with CD and written materials as format options. Additional suggestions included broadening access of professionals in rural areas and of students.

## Discussion

Of online-CE courses identified by systematic reviews[21], [22], not many in Latin America and Africa have provided comprehensive I-CE and to our knowledge, few were developed as case-based training within in a developing country[23], [24].

We found that an Internet-based CE course implemented in 10 cities in Peru was feasible, well accepted by physicians and midwives and improved knowledge that was sustained at four month follow-up.

Using local technology, minimal resources (post-doctoral trainee salary for FC for 12 months, plus approximately $5,000 for course production, including platform, content, and field work), and evidence-based strategies, we provided the course to nearly half of the physicians and midwives working in the private sector in 10 of the larger cities throughout Peru. Evaluation has provided evidence for impact on knowledge for those who took the course, but not for those who enrolled but did not actually take the course; but no impact even among participants on self- reported practice.

Rates of recruitment, participation, and follow-up testing, and the diversity of participation in our study were substantially higher than what is generally considered achievable for this type of CE[25]. Recruitment and participation of physicians has reportedly been challenging for educational interventions. An online course on chlamydia screening reported recruitment of 33% of physicians and rates of participation of those recruited to be about 52%[25]. Rates of participation in our study likely reflect high level of interest in this topic and in Internet-based CE per se, lack of alternative sources of CE, and feasibility of internet-based training throughout the country.

Acceptability of the course was reflected not only by high participation rates, but by satisfaction with the course as expressed in the post-test survey, with96% of participants rating the course as very useful and relevant to their clinical practice. Although response bias can explain high satisfaction rates, similar web-based studies have found rates as low as 47% for physicians' perceptions about course relevance to their clinical practice[26].

Improvements in knowledge were greatest among females, those who participated in the pre-course workshops, those who actually took the course, those with lower scores at baseline, and those not already PREVEN Network members. The beneficial effect of the pre-course workshop could reflect either an effect of in-person participation in the pre-course workshop on subsequent internet-based education, or selection bias (motivated participants could have taken the course and then performed better). The limited improvement in knowledge of PREVEN Network members could indicate a ceiling effect of this particular training program; information provided by this Internet course overlapped with that provided to PREVEN Network members a year earlier. It is conceivable that some of the improvement in scores could be explained by a direct *per se* effect of taking the pre-course test, or could represent a regression to the mean[27]. The last is a common criticism of evaluation of educational interventions without use of control groups[28]. However non-course users did not significantly improve their test scores, consistent with an effect of the course.

The significant and sustained improvement from pre- to post- to follow-up test in knowledge scores could be attributable to some elements of our course identified in randomized trials of other well-designed educational web-based programs[10], [29], [30], [31], [32], [33]. The course design used a learning theory approach, was based on participants' needs-assessments, had an interactive format, used case-based learning with performance feedback, and was tailored to local STD problems and included reinforcement components (e.g., mail consultation, and learning materials).

Although we attempted to avoid features commonly found and criticized in traditional I-CE[3], [34], [35], additional elements also shown to be effective were not used in this intervention. These elements include illustrative dynamic schemes and flow charts; use of slides synchronized with audio; didactic presentations using video; live Web conferences; online risk assessment calculators; e-mail reminders; asynchronous discussions with peers and facilitators; chat rooms; telephone contacts; game-like simulations; and other novel elements including computerized virtual patients for mobile phones. Reviews and meta-analysis of studies assessing impact of Internet-based courses have concluded that they are comparable or better than face-to-face courses[22], [29], and that a combination of course formats (on line and in class) are more effective than face-to-face courses[36].

In contrast to the overall improvement of test scores with our course, no significant improvement was noted in self-reported practices. Moreover a significant decrease in the provision of referral cards for partners' treatment was reported by midwives but not by physicians. The lack of reported improvement in practices could be explained by the high rates of correct practices reported at baseline, possibly related to the social desirability response bias. Other explanations could be self-selection of participants into the course (e.g., about 60% were already PREVEN Network members); lack of validity of the measure for assessing practice; or no actual translation of knowledge into practice. Translation of knowledge into practice is a complex process that is not always achieved[37]. Although partner referral cards were available on the course web site, the low rate of using referral cards for partner treatment suggests the need to establish a better enabling system to complement this training, and deserves further research on how to promote partner notification.

This was not a randomized controlled trial, and results are therefore subject to bias. Several factors were plausibly associated with score improvement in knowledge and with feasibility and acceptability of the implementation. Beyond the lack of a control group, a limitation of our study is that we did not measure objective changes in practices or in patients' outcomes. We measured changes in self-reported practices, not a highly reliable measurement^.^ Self-reported measures are susceptible to biases (tendency to answer positively, give more socially acceptable answers, etc.). Another study limitation is the validity and reliability of knowledge assessments by questionnaires, which can be questioned because of the absence of standardization and difficulty when comparing them across studies[38], [39], [40]. Novel methods, such as computerized clinical vignettes, to assess knowledge and clinical performance in a variety of medical areas are more often being used and studied[29], [40]. Extrapolation of the study results may be done with caution to settings other than that represented by the study participants. However, inclusion of samples from varied setting - coastal, jungle, and Andean region does allow a wider generalization. Further studies are needed to explore the utility of I-CE courses in other resource restricted settings for broader applicability.

### Conclusions

With rapidly growing access to the Internet in much of the developing world this study provides evidence that an I-CE course is feasible, acceptable, and attracts a high level of interest. I-CE has great potential to improve the training of health care workers and to reduce information gaps in developing country settings. Future research should include randomized controlled trials comparing different types of I-CE or instructional elements; cost effectiveness studies; and development and evaluation of instruments to measure clinicians' performances. In the long-term, creating comprehensive online training centers could be used to coordinate Internet-based curricula between universities, affiliated institutions, and the public sector in developing countries.

## Supporting Information

Appendix S1 Example of case-based clinical vignette and sequential questions.
(DOC)

Appendix S2 Internet-based CE Course Images: Illustration of computer screens showing Internet-based CE modules used in the Intervention.
(PDF)
