# Supplementary material for: An Interactive Internet-Based Continuing Education Course on Sexually Transmitted Diseases for Physicians and Midwives in Peru
Source: PLoS One. 2011 May 9;6(5):e19318. doi: 10.1371/journal.pone.0019318 (PMC3090386; doi:10.1371/journal.pone.0019318)
Supplement: Appendix S1 — Example of case-based clinical vignette and sequential questions. (DOC) [file pone.0019318.s001.doc]

| **TREATMENT OF URETHRAL DISCHARGE**  In the previous case, Julio, a 20 year old patient, reported as principal symptoms burning on urination and presence of yellow spotting on his underwear. During the clinical examination, there was not evidence of urethral discharge. However, after asking the patient to “milk the discharge forward”, a spontaneous small amount of purulent yellow secretion was observed coming from the meatus. (Figure 1) The rest of the physical examination was normal.  You explain to your patient that he has urethritis, a sexually transmitted disease.  **According to the Peruvian clinical national guidelines or the WHO guidelines, which of the following is the treatment for the urethral discharge syndrome?**  A. Gentamicin 3-6 mg/kg. IM q 8 hours for 7 days.  B. Spectinomycin 2 g in a single IM dose.  C. Benzathine penicillin G 2.4 million units IM in a single dose.  D. Doxycycline 100 mg orally twice a day (q 12 hours) for 7 days.  E. Ciprofloxacin 500 mg orally in a single dose plus Azithromycin 1 g orally in a single dose.  **Example of answers feedback**  **A. Gentamicin 3-6 mg/kg of body weight every 8 hours IM for 7 days.**  **THIS IS A WRONG ANSWER**  Urethritis (urethral inflammation) is produced by infectious and noninfectious conditions and may be symptomatic or asymptomatic. Symptoms include mucopurulent or purulent discharge, dysuria, or urethral pruritus.  Since most common infectious causes of urethritis includes *N. gonorrhoeae* and *C. trachomatis*, Julio should be treated for both gonorrhea and chlamydia.  Gentamicin, an aminoglycoside, has no effect against *C. trachomatis* and is not a first line drug for the treatment of *N. gonorrhoae*. Therefore this treatment is not correct for Julio.    According to a cross sectional study on physicians in private practice in Peru (1), over a period of 15 years (1983-1997), treatment of urethral discharge was frequently inadequate due to:  * Inadequate coverage for chlamydia.  * Frequent election of second line drugs.  In this cross-sectional study aminoglicocides such as amikacin and kanamicin were drugs commonly prescribed by physicians in private practice for urethral discharge.  (1) Garcia PJ, Holmes KK. STD trends and patterns of treatment for STD by physicians in private practice in Peru. Sex Transm Infect 2003;79(5):403-7.  **PLEASE CHOOSE ANOTHER ANSWER** |
| --- |
