# Supplementary material for: An Interactive Internet-Based Continuing Education Course on Sexually Transmitted Diseases for Physicians and Midwives in Peru
Source: PLoS One. 2011 May 9;6(5):e19318. doi: 10.1371/journal.pone.0019318 (PMC3090386; doi:10.1371/journal.pone.0019318)
Supplement: Appendix S2 — Internet-based CE Course Images: Illustration of computer screens showing Internet-based CE modules used in the Intervention. (PDF) [file pone.0019318.s002.pdf]

Appendix S2.1 Course home page.

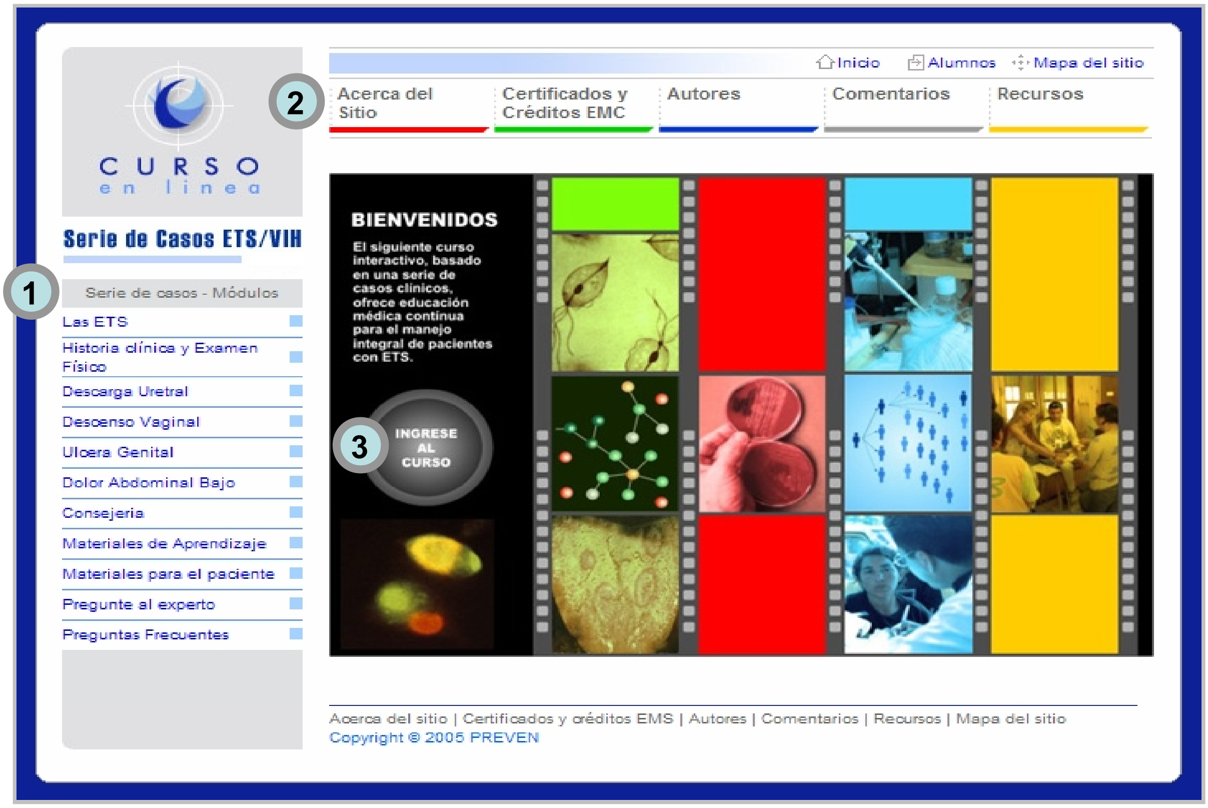

1. Modules
   STD overview
   History taking and physical examination
   Counseling regarding risk behavior
   Provided learning materials
   Materials for the patient
   Opportunities to “ask the expert”
   Responses to frequently asked questions
   Links to educational resources and evidence-based content
2. Start button
3. Main menu

Appendix S2.2. Instructional elements of the Internet-based CE course.

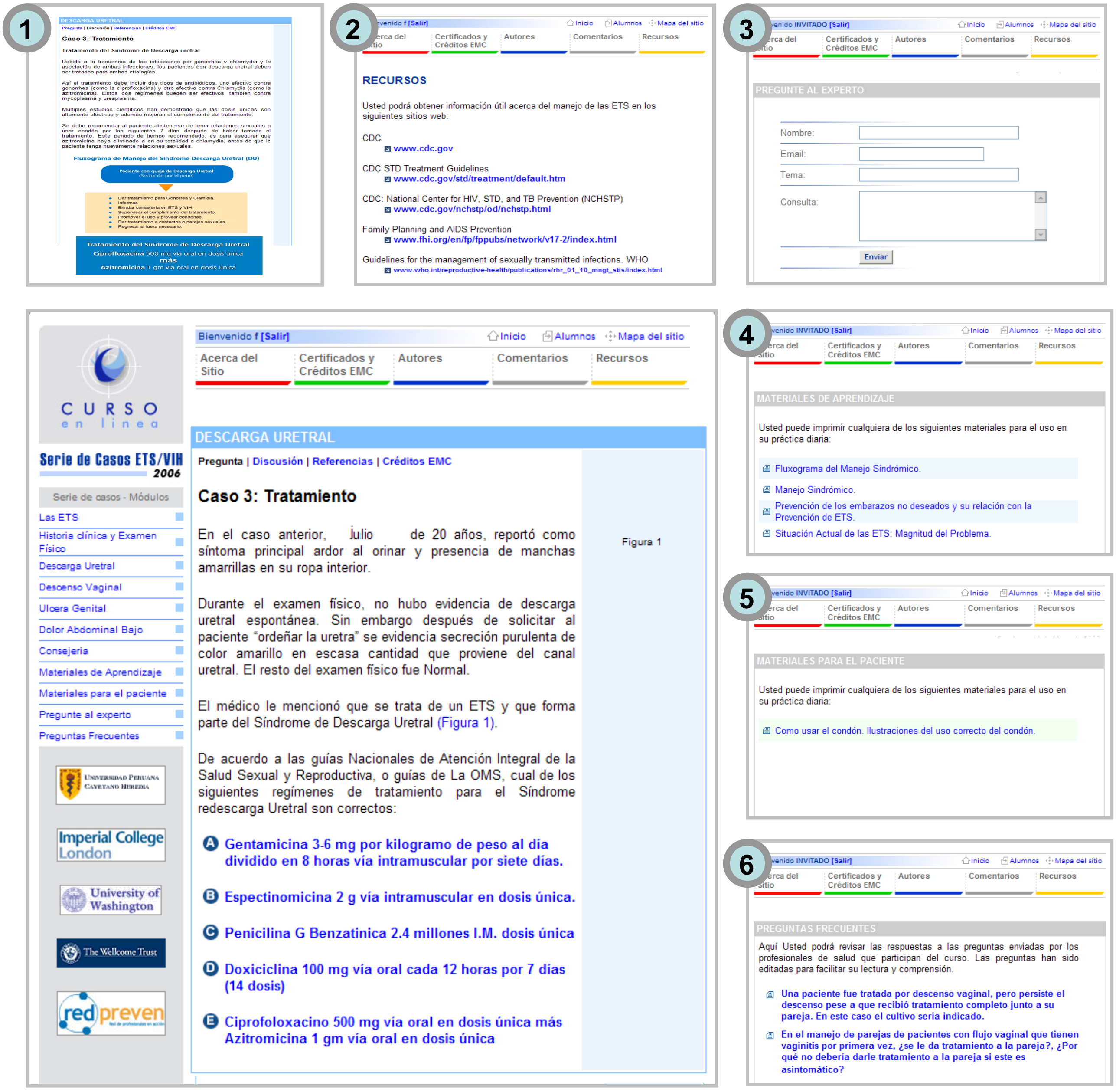

1. In depth discussion
2. Resources
3. Asking the expert
4. Learning materials
5. Materials for the patient
6. Frequently asked questions

Note: Further feedback and educational support were provided through post-course consultations via e-mail and summarized in the responses to frequently asked questions module.
